# Hospital daily outpatient visits forecasting using a combinatorial model based on ARIMA and SES models

**DOI:** 10.1186/s12913-017-2407-9

**Published:** 2017-07-10

**Authors:** Li Luo, Le Luo, Xinli Zhang, Xiaoli He

**Affiliations:** 1 Business School, Sichuan University, No. 29, Wangjiang Road, Chengdu, Sichuan 610064 China; 2 Outpatient Department, West China Hospital, Sichuan University, Chengdu, Sichuan 610064 China

**Keywords:** Daily outpatient visits, ARIMA, SES, Combinatorial forecasting model

## Abstract

**Background:**

Accurate forecasting of hospital outpatient visits is beneficial for the reasonable planning and allocation of healthcare resource to meet the medical demands. In terms of the multiple attributes of daily outpatient visits, such as randomness, cyclicity and trend, time series methods, ARIMA, can be a good choice for outpatient visits forecasting. On the other hand, the hospital outpatient visits are also affected by the doctors’ scheduling and the effects are not pure random. Thinking about the impure specialty, this paper presents a new forecasting model that takes cyclicity and the day of the week effect into consideration.

**Methods:**

We formulate a seasonal ARIMA (SARIMA) model on a daily time series and then a single exponential smoothing (SES) model on the day of the week time series, and finally establish a combinatorial model by modifying them. The models are applied to 1 year of daily visits data of urban outpatients in two internal medicine departments of a large hospital in Chengdu, for forecasting the daily outpatient visits about 1 week ahead.

**Results:**

The proposed model is applied to forecast the cross-sectional data for 7 consecutive days of daily outpatient visits over an 8-weeks period based on 43 weeks of observation data during 1 year. The results show that the two single traditional models and the combinatorial model are simplicity of implementation and low computational intensiveness, whilst being appropriate for short-term forecast horizons. Furthermore, the combinatorial model can capture the comprehensive features of the time series data better.

**Conclusions:**

Combinatorial model can achieve better prediction performance than the single model, with lower residuals variance and small mean of residual errors which needs to be optimized deeply on the next research step.

## Background

Due to a rapid growing aging population, medical resources in China are comparatively scarce, with disproportional distribution and generally insufficient medical investment. It causes a great gap between supply and demand, as well as the increasing attention on optimal management of healthcare resources, which makes accurate forecasts of future healthcare demand and resource availability more significant and critical. Outpatient department (OD) is the window of the hospital external service from the hospital actual operation, and is experiencing increasing stress from year to year because of the increasing patient volumes, the growing complexity of health conditions and so on. The ability to predict outpatient visits is crucial for resource planning and allocation as well as efficient appointment scheduling in OD aimed to avoiding overcrowding and providing high quality patient care service [1]. Moreover, as the key and major element to implement the hospital management, daily outpatient visits are directly related to the workload of medical examinations and hospitalization services. Precise and reliable forecasts of the different types of outpatients amount can contribute to allocate the key healthcare resources scientifically (such as medical equipment, surgical equipment and hospital beds). So that it is significant to make an accurate forecast for the daily outpatient visits in advance, in order to help hospital managers to make the right decisions to meet the expected healthcare demand effectively and timely.

In the field of health care system, increasing attention has been paid to the prediction of patient arrivals in hospital establishments, especially the prediction of Emergency department(ED)visits or admission which is substantially differ from the outpatient visits prediction [2–5]. According to ED data,patients arrive in an unplanned fashion beyond the control of the hospital, and the number of ED visits shows strong periodical, seasonal and stochastic fluctuations driven by factors such as climate, epidemic disease, the type of the day and socio demographic effects [6–8]. Based on these features, researchers used different methods to predict the number of ED visits hourly, daily and monthly [5, 6, 9]. In contrast, mainly affected by the current healthcare system, China’s outpatient visit is more of a planned elective nature. Patients can self-refer to any provider they could afford when they feel sick, and they should be booked days or weeks in advance to seek outpatient service in most of large-scale hospitals. However, in light of the fixed team arrangement and the limited outpatient service resources, the hospital can only accept a certain amount of outpatient visits every day, and the number of OD visits in large-scale hospitals fluctuates much less violently on a weekly, monthly or yearly basis. Instead, the daily number of OD visits shows a periodic fluctuation on a weekly basis, with a strong day-in-week variation mostly attributed to the doctors’ influence, which can be transferred to the downstream service nodes and have much influence on preparing the required reserve capacity in all sectors of hospital. Therefore, accurate prediction of the daily outpatient visits in 1 week can contribute to reasonable formulation of the weekly rotating shift schedule, which is of high importance to achieve an efficient routine management of outpatient services in hospitals. Meanwhile, it is also crucial for administrators in other sectors to optimize resource allocation and strategic planning.

Motivated by overcrowding and resource scheduling problems arising in healthcare systems in China, we choose a typical hospital-West China Hospital (WCH) as cooperated hospital, and consider a time-series forecasting problem of daily outpatient visits. WCH is a public tertiary teaching hospital in Chengdu, China, which has large-scale outpatients. It served about 4.9 million outpatients and emergency attendances in 2014, and the number of outpatient visits accounted for about a quarter of the total amount. Research shows the amount of outpatient attendance and medical service needs are significantly affected by patient’s disease type, distance to healthcare providers and other factors, which present high level varying characteristics [10]. It’s worth noting that Chengdu patient accounts for the largest proportion of outpatients by each specialty of WCH. According to its internal statistical analysis on outpatient amount in 9 common and main specialty care units, the proportion of outpatients living in Chengdu city reached 46.31% in 2014.Compared with patients from other cities of Sichuan province and those from other provinces, the arrival pattern of outpatients from Chengdu showed higher randomness, moreover, the daily outpatient volume fluctuated relatively frequently. Therefore, this paper chose two common disease types (Endocrine and Respiratory disease) as illustrations to study the daily volume prediction of Chengdu outpatients.

### Literature

For the past few years, increasing attention has been accorded to time series models to predict demands for medical services, such as patient arrivals, hospital discharges and length of stay [2, 3]. Compared to another classical method of regression, time series model [4, 5] is important for healthcare managers to capture features of short-term fluctuations better [3]. Being a general class of linear model, the ARIMA model can perfectly capture linear patterns in a time series with minimum computational efforts, so most studies adopt them to describe the relationship between the variables or use them as the benchmark to test the effectiveness of combined models with mixed results [9]. Sun et al. [11] and Kadri et al. [1] developed ARIMA models for forecasting daily attendances at ED of hospitals to prove time-series analysis to be a useful, readily available tool for predicting ED workload. Li et al. adopted ARIMA model to forecast monthly outpatient visits in a general hospital in China [9]. However, relationship between target variable and factors in many time series is nonlinear and complex, many recent studies have focused on the use of machine learning techniques as alternatives to the traditional time series methods, such as Fuzzy logic, Artificial Neural Network(ANN), Genetic Algorithms, Support Vector Machine(SVM), Taylor expansion and non-parametric smoothing etc. [1, 12–14]. Cheng et al. [15] and Garg et al. [12] proposed new fuzzy time series methods to forecast the number of outpatient visits. Hadavandi et al. [1] presented a hybrid artificial intelligence model based on Genetic Fuzzy Systems to forecast monthly outpatient visits of the department of internal medicine with high accuracy. Wang et al. [4] developed a novel forecasting model based on hybridization the firefly algorithm and support vector regression to forecast the daily diarrhoeal outpatient visits in Shanghai. Moreover, because a real-world time series is complex in nature, and a single linear or nonlinear model may not be totally sufficient to identify all the characteristics of the time series datasets, so many hybrid or combined models have been proposed to complement each other to improve forecasting accuracy while the literature on this topic has expanded dramatically [1, 16, 17]. Although machine learning techniques based on data-driven and non-parametric models could outperform many traditional techniques, they also have some limitations, such as unknown or hard to describe the underlying relationships among datasets [15]. As we known, high prediction accuracy depends not only on modeling but also on understanding of the data features, which means that advanced, sophisticated, and simpler extrapolation methods must be associated with specific features of data [18]. Hence, this paper differs from previous studies, in which we attempt to simplify the outpatient visits prediction problem and improve the forecasting accuracy by capturing the intrinsic fluctuating characteristics of the hospital’s daily outpatient visits.

The analysis and forecasting of hospital daily outpatient visits belongs to time series prediction problem, in which the features of randomness and cyclic fluctuations have the biggest effect on forecasting accuracy [18]. Due to the ability of effectively extracting the linear features of randomness and trend, ARIMA model has found extensive applications in forecasting hospital daily visits of ED and OD [6, 19, 20]. Furthermore, aiming to better capture cyclicity over a period of week in daily time series data, ARIMA models have been extended and modified among which seasonal ARIMA (SARIMA) and multiplicative seasonal ARIMA (MSARIMA) models are the most widely used [3, 7, 21]. In addition to randomness and periodicity, the day of the week effect is also present in daily volume of patients in ED and OD as well as daily number of discharged patients [3, 8], which refers to the situation where the time series at the same time point within 7 days a week during different weeks show non-linear trend variation and some change patterns can be found in the data from Monday to Sunday. Several researchers proposed seasonal regression model to capture the day of the week effect of time series data, but it may be problematic and lead to poor forecasts due to the nonlinear patterns. Because of the good performance in capturing the nonlinear pattern and description of both trend and seasonality in the data, exponential smoothing(ES) methods have since been among the most widely used forecasting procedures in industry and commerce as well as healthcare [7, 22, 23]. Automated ES approach [5] and simple seasonal ES model [7] were applied to forecast the demand of medical care in terms of monthly and daily visits in ED of hospitals. Previous study indicated that the ES model incorporated the time correlation knowledge, made full use of the historical information [16] and consequently yielded superior performance in modeling ED demand due to its simplicity, robustness and accuracy [7]. Moreover, it was also demonstrated that although model selection should be done individually (per series) when we deal with heterogeneous data as to capture the different features met in each series [18], forecasting models alone are not sufficient for decision making [24], and combining models could improve predictive accuracy [25]. While ES model which is based on a description of nonlinear feature of trend and seasonality and SARIMA model which aims to capture the linear feature of autocorrelations and periodicity in the data provide complementary approaches to the prediction problem, we try to develop a new model to forecast the daily number of OD visits by combing them.

This paper examines the issue of forecasting the daily outpatient visits in China’s large hospitals which are often overloaded with various types of patients. The objective is to develop a methodology, present data and modeling details and provide results of modeling the daily outpatient visits. In order to achieve good forecasting performance, several time series models are built for different features in each series respectively, and a combinatorial model is obtained for short-term forecasting. First we construct a SARIMA model to capture the cyclicity and autocorrelation in daily time series data. In terms of non-linearity in weekly time series data of the same day over different weeks, single exponential smoothing (SES) model is adopted to treat the day of the week effect. After that, we establish a linear combinatorial model based on the above models through residual modification approach. Finally the combinatorial forecasting model is verified by empirical studies involving two time series data of Chengdu outpatients in departments of endocrinology and respiratory in WCH over the entire year of 2014. The daily outpatient visits are predicted about 1 week ahead using the proposed model, and the results indicate that the combinatorial model outperforms any of the above two single traditional models.

## Methods

In this section, we describe several models for forecasting the daily outpatient visits. Two models on time series are built for different traits of the outpatient visit data, and the combinatorial forecasting model is established based on the residual modification idea:

1 $$ {\mathrm{X}}_T={{\mathrm{l}}_1}^{\ast }{\mathrm{X}}_T^1+{{\mathrm{l}}_2}^{\ast }{\mathrm{X}}_T^2 $$

Where ${\mathrm{X}}_{\mathrm{T}}^1$ represents the estimate value of daily outpatient visit for daily time series data, which is estimated by SARIMA model in “Seasonal ARIMA model” section; ${\mathrm{X}}_{\mathrm{T}}^2$ represents the estimate value of daily outpatient visit for the day of the week time series (weekly time series) data, which is estimated by SES model in “Single exponential smoothing model” section; l_i_ is the weighting coefficient of the single model i, calculated as describe in “Combinatorial model based on SARIMA and SES” section.

### Seasonal ARIMA model

ARIMA model is an extension of ARMA model, combining differential operation and ARMA model. Seasonal ARIMA model is to model on the time series presenting with obvious seasonal effect (cyclic effect), by smoothing over the time series through trend and seasonal differencing along with the fitting of ARMA model. Its theoretical model has the following form:

2 $$ \upvarphi \left(\mathrm{B}\right){\nabla_{\mathrm{D}}\nabla}^{\mathrm{d}}{\mathrm{x}}_{\mathrm{t}}^1=\Theta \left(\mathrm{B}\right){\upvarepsilon}_{\mathrm{t}} $$

where ${\mathrm{x}}_t^1$ and *ε*_*t*_ are the observed value of hospital daily outpatient visits and random error at time period t, D is step length for single cycle, d is order of differential equation for extracting trend and B is backshift operator. $\upvarphi \left(\mathrm{B}\right)=1-{\upvarphi}_1\mathrm{B}-{\upvarphi}_2{\mathrm{B}}^2-\dots -{\upvarphi}_{\mathrm{p}}{\mathrm{B}}^{\mathrm{p}}$ and $\Theta \left(\mathrm{B}\right)=1-{\uptheta}_1\mathrm{B}-{\uptheta}_2{\mathrm{B}}^2-\dots -{\uptheta}_{\mathrm{q}}{\mathrm{B}}^{\mathrm{q}}$ are the polynomials in B for AR and MA components, respectively.

Four steps are involved for modeling and forecasting using SARIMA model: smoothing over time series (differential operation), model recognition, parameter estimation and model diagnosis, and model forecasting. In the smoothing step, data transformation is often needed to make the time-series stationary. Results or plots of autocorrelation function (ACF) and partial autocorrelation function (PACF) of the sample data are the basic tools for analyzing stationary and order of model. When trend and seasonality are observed in the time series, differencing and log transformation are applied to eliminate the fluctuation to stabilize the variance. Once the initial model is determined, the key difficulty is the estimation of the other model parameters specifically, which can be estimated by an optimization procedure according to the Bayesian information criterion (BIC). To recognize whether the chosen model can describe the fluctuation pattern of time series data well, goodness-of-fit test is needed mainly through white noise test. If the residual series is not a white noise sequence, it is necessary to repeat above steps to create a new model.

### Single exponential smoothing model

To deal with non-linearity in moving average interval, larger weights are assigned to short-term observations, while smaller weights to long-term observation, so as to achieve better forecasting result. Time series of daily outpatient visits of the same day in different weeks are modeled using SES model, and its general form is as follows:

3 $$ \mathrm{x}\widehat{{}_{t^{\prime }+1,\uptau}^2}={\upalpha \mathrm{x}}_{t^{\prime },\uptau}+\left(1-\upalpha \right)\mathrm{x}\widehat{{}_{t^{\prime },\uptau}^2} $$

where α is smoothing constant, ranging from 0 to 1; ${\mathrm{x}}_{t^{\prime },\uptau\ }$ is the observed value of daily outpatient visits of day τ in week t^′^; $\widehat{{\mathrm{x}}_{t^{\prime },\uptau}^2}$ is the smoothed value of daily outpatient visits of day τ in week t^′^ and $\widehat{{\mathrm{x}}_{t^{\prime }+1,\uptau}^2}$ is the smoothed value of daily outpatient visits of day τ in week t^′^ + 1, which is selected to be the predicted value. The optimal value of α is found using an optimization method according to the criteria of minimum values of mean square error (MSE) and mean absolute percentage error (MAPE) between the predicted and observed values. The means of the observed values in the first 3 weeks in the same time series is taken as the initial value $\widehat{{\mathrm{x}}_{1,\uptau}^2}$ for SES forecasting [25].

### Combinatorial model based on SARIMA and SES

#### Combinatorial model

In order to better capture the characteristics of autocorrelation, cyclicity and the day of the week effect of daily outpatient visits, a combinatorial model based on the two above models is built through residual modification idea in the form of weighted averaging. The formulations can be written as:

4 $$ \mathrm{X}\widehat{{}_{{\mathrm{t}}^{\prime },\uptau}}={{\mathrm{l}}_1}^{\ast}\widehat{{\mathrm{x}}_{\mathrm{t}}^1}+{{\mathrm{l}}^2}^{\ast}\widehat{{\mathrm{x}}_{{\mathrm{t}}^{\prime },\uptau}^2} $$

5 $$ \mathrm{t}=7\times \left({\mathrm{t}}^{\prime }-1\right)+1 $$

where $\widehat{{\mathrm{x}}_{\mathrm{t}}^1}$ and $\mathrm{x}\widehat{{}_{t^{\prime },\uptau}^2}$ are the estimated values of daily outpatient visits from SARIMA and SES, respectively; $\mathrm{x}\widehat{{}_{{\mathrm{t}}^{\prime },\uptau}}$ is the predicted value of the weighted combinatorial model; and l_i_ is weighting coefficient of single model i which is formulated as

6 $$ {\ \mathrm{l}}_{\mathrm{i}}=\frac{{{\mathrm{E}}_{\mathrm{i}}}^{-1}}{\sum_{\mathrm{i}=1}^2{{\mathrm{E}}_{\mathrm{i}}}^{-1}},\mathrm{i}=1,2 $$

**Fig. 1:**
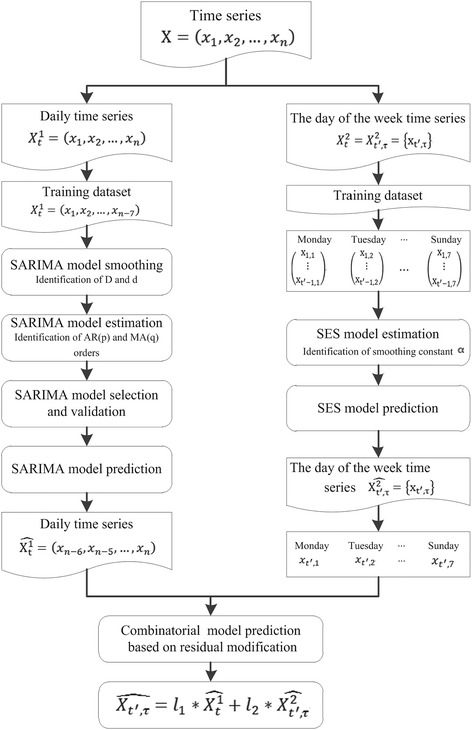
The framework of proposed combinatorial model based on SARIMA and SES models

Where l_1_ + l_2_ = 1, and ${\mathrm{E}}_{\mathrm{i}}=\sqrt{\sum_{\mathrm{i}=1}^{\mathrm{n}}{{\mathrm{e}}_{\mathrm{i}}}^2}$. The weighing coefficient is determined by the error indicators of the single model, and *E*_*i*_ is the error variance sum of the single model which is the indicator of forecasting accuracy. The lower the E_i_, the higher the forecasting accuracy of model i and hence the higher the importance in combinatorial forecasting model. And, the framework of proposed combinatorial model based on SARIMA and SES models is shown in Fig. 1.

#### Testing indicator

MAPE is used as the indicator of forecasting accuracy, calculated as

7 $$ \mathrm{MAPE}=\left(\sum |\frac{{\mathrm{x}}_{\mathrm{t}}-\widehat{{\mathrm{x}}_{\mathrm{t}}}}{{\mathrm{x}}_{\mathrm{t}}}|/\mathrm{n}\right)\times 100\% $$

## Results

Daily visits data of Chengdu outpatients in departments of endocrinology and respiratory in WCH over the year of 2014 are used for forecasting the daily outpatient visits 1 week ahead. In 2014, the total number of endocrinology outpatient visits (EOV) in WCH was 120,107, 57,763 of which came from Chengdu, accounting for 48.01%, while the total number of respiratory outpatient visits (ROV) was 108,045, 45,629 of which came from Chengdu, accounting for 42.23%.

### Preliminary data analysis

**Fig. 2:**
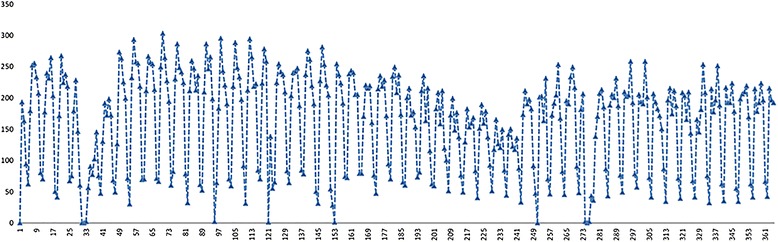
Daily time-series data of EOV in 2014

**Fig. 3:**
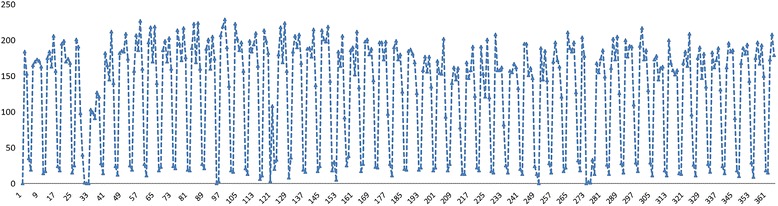
Daily time-series data of ROV in 2014

Daily visits data of Chengdu outpatients in departments of endocrinology and respiratory in WCH over the year of 2014 are used respectively, each totaling 365. The data are inputted into Excel 2010 and SAS 9.4. Time series charts are generated for data from January 1st to December 31st, 2014. The average number of EOV and ROV are about 158/day and 125/day respectively, and the standard deviations are 77.87 and 76.11 respectively, which might mainly result from large fluctuations in seasonal effects and special day effects, such as holidays and weekends. Figures 2 and 3 provide a first view of the data. It can be seen that the daily outpatient visits fluctuate greatly, especially on holidays and in summer and winter vacation. Due to summer and winter vacation and the Spring Festival holiday, the outpatient visits in February and August are the lowest throughout the year. Moreover, a cyclic variation pattern is found over the period of a week, while great differences exist among different points in a same cycle, such as fewer outpatient visits over the weekends.

To account for the cyclicity, daily outpatient visits data spanning over incomplete periods are removed. Therefore, the time series from week 2 to week 52 (totally 51 weeks) are used for model building. The models are fitted for the first 301 observations from week 2 to week 44 (*N* = 301, 84% of all data as training set, from January 1, 2014 to November 2, 2014). The remaining 56 observations from week 45 to week 52 are utilized for examining the forecasting ability of fitted models (*T* = 56, 16% as testing set, from November 3, 2014 to December 28, 2014). That is, time series of 43 weeks for model fitting and time series of 8 weeks for verification while predicting each week of last 2 months based on all observations 1 week before. We explain the process of how to predict the daily outpatient visits in the 45th week of time series in detail, and so on, for each of the rest 7 weeks.

### Pre-processing of singularities on holidays

The daily outpatient visits present obvious fluctuations in weekly pattern and special day effect, and the decrease of visits during national holidays such as the Spring Festival, the May Day, the Mid-Autumn Festival, etc. are significant, mainly resulting from the patients’ traditional customs and behavior habits. They can’t real reflect the daily routine fluctuations of outpatient visits. Therefore, singularity is identified which is distributed outside of the scope of 2 times the standard deviation from the mean value in each the day of the week time series. Finally, ten singularities in time series data of EOV as well as twelve in time series data of ROV are picked out and pre-processed, by replacing them with the mean of the same day in the previous and the following weeks.

### Building the seasonal ARIMA model

#### Data analysis and differential operation

**Fig. 4:**
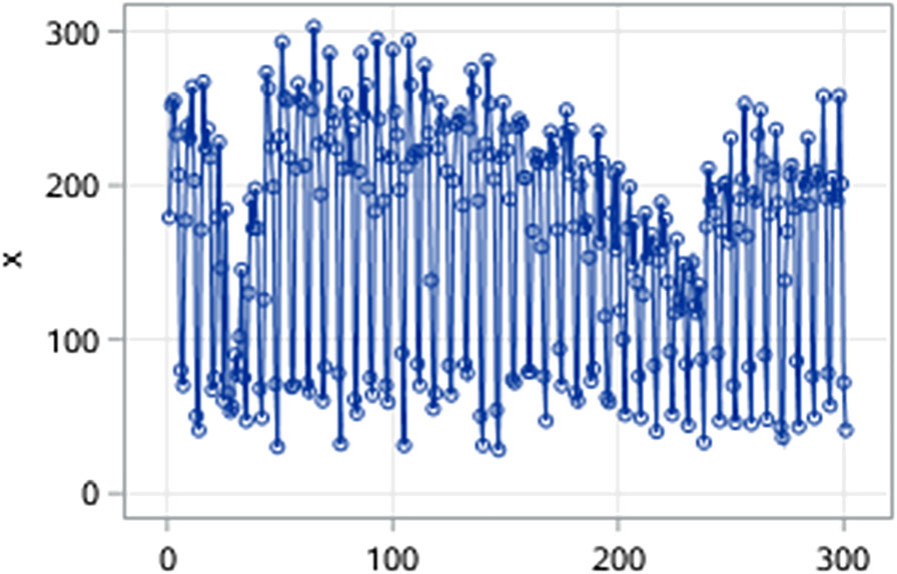
Time series data of EOV

**Fig. 5:**
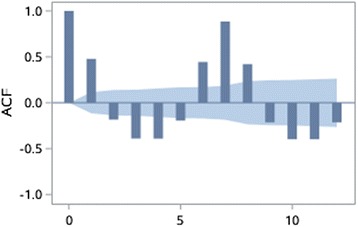
Autocorrelation coefficients of the original time series of EOV

**Fig. 6:**
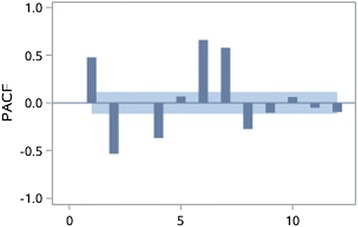
Partial autocorrelation coefficients of the original time series of EOV

**Fig. 7:**
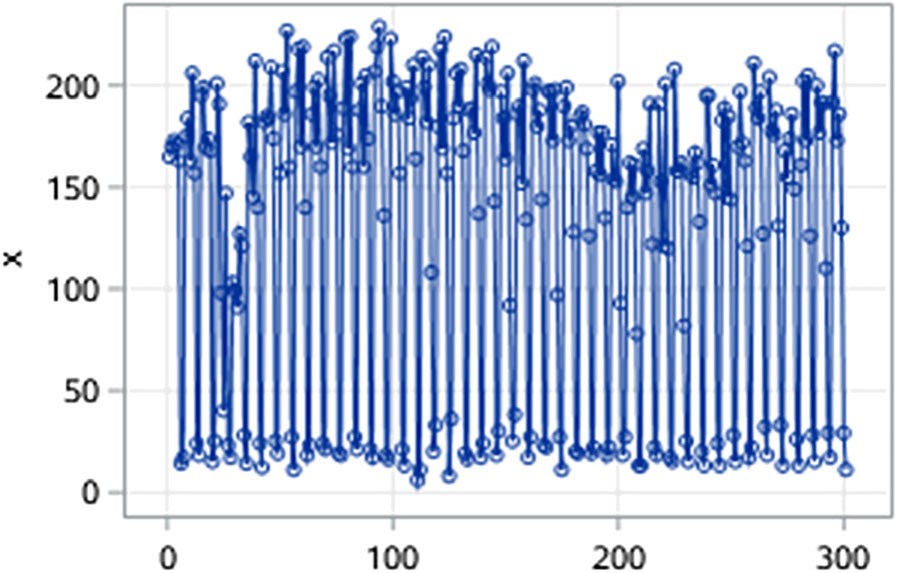
Time series data of ROV

**Fig. 8:**
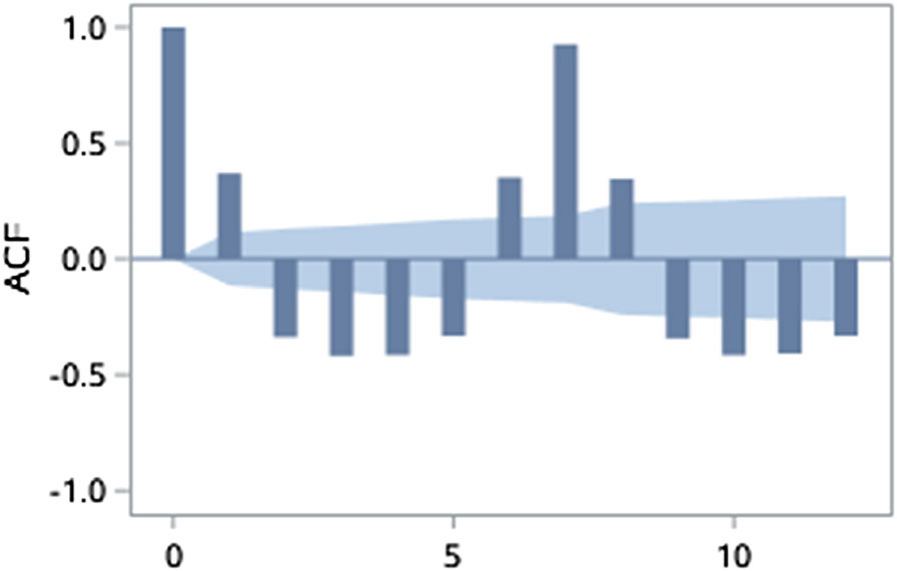
Autocorrelation coefficients of the original time series of ROV

**Fig. 9:**
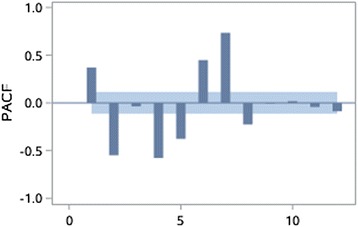
Partial autocorrelation coefficients of the original time series of ROV

The stationarity of the time series is assessed through autocorrelation chart (Figs. 4, 5, 6, 7, 8, 9). The time series of EOV and ROV are non-stationary sequences and display strong cyclicity feature over the period of 7 days. Deterministic information is extracted from the original time series in these two departments both using first-order 7-step differencing. And the differential time series are verified as non-random series through white noise test. Then ARMA models are fitted and used for forecasting.

#### Model recognition and parameter estimation

According to BIC, the optimal model is the one with the minimum value of BIC function, which is ARIMA (1,1,7) model for EOV and ARIMA(2,1,7) model for ROV. Model parameters are estimated with least-squares estimation and the parameters failing to pass the significance test are further adjusted. The estimated parameters are shown in Tables 1 and 2. According to residual analysis, *P* value of Ljung-Box test statistics at all lags is more than 0.05, indicating the reliability of the model design.

**Table 1:** Parameter estimation and testing of EOV

Parameter	Estimation	Standard error	t value	Approximate Pr > |t|	Lag
MA1,1	0.45393	0.05165	8.79	<.0001	2
MA1,2	0.25916	0.04457	5.81	<.0001	3
MA1,3	−0.14116	0.04477	−3.15	0.0018	6
MA1,4	0.42807	0.04878	8.78	<.0001	7
AR1,1	−0.51364	0.05550	−9.25	<.0001	1

**Table 2:** Parameter estimation and testing of ROV

Parameter	Estimation	Standard error	t value	Approximate Pr > |t|	Lag
MA1,1	0.30118	0.04524	6.66	<.0001	2
MA1,2	0.36505	0.04345	8.40	<.0001	3
MA1,3	−0.14669	0.04209	−3.49	0.0006	5
MA1,4	−0.21579	0.03792	−5.69	<.0001	6
MA1,5	0.65310	0.03797	17.20	<.0001	7
AR1,1	−0.68602	0.05706	−12.02	<.0001	1
AR1,2	−0.31708	0.06558	−4.83	<.0001	2

The model forecasting the EOV is fitted as

8 $$ {\nabla}_{\mathrm{D}}{\nabla}^{\mathrm{d}}\widehat{{\mathrm{x}}_{\mathrm{t}}^1}=\frac{\left(1-0.45393{\mathrm{B}}^2-0.25916{\mathrm{B}}^3+0.14116{\mathrm{B}}^6-0.42807{\mathrm{B}}^7\right){\upvarepsilon}_{\mathrm{t}}}{1+0.51364\mathrm{B}} $$

and the model forecasting the ROV is fitted as

9 $$ {\nabla}_{\mathrm{D}}{\nabla}^{\mathrm{d}}\widehat{{\mathrm{x}}_{\mathrm{t}}^1}=\frac{\left(1-0.30118{\mathrm{B}}^2-0.36505{\mathrm{B}}^3+0.14669{\mathrm{B}}^5+0.21579{\mathrm{B}}^6-0.65310{\mathrm{B}}^7\right){\upvarepsilon}_{\mathrm{t}}}{1+0.68602\mathrm{B}+0.31708{\mathrm{B}}^2} $$

#### Forecasting

**Fig. 10:**
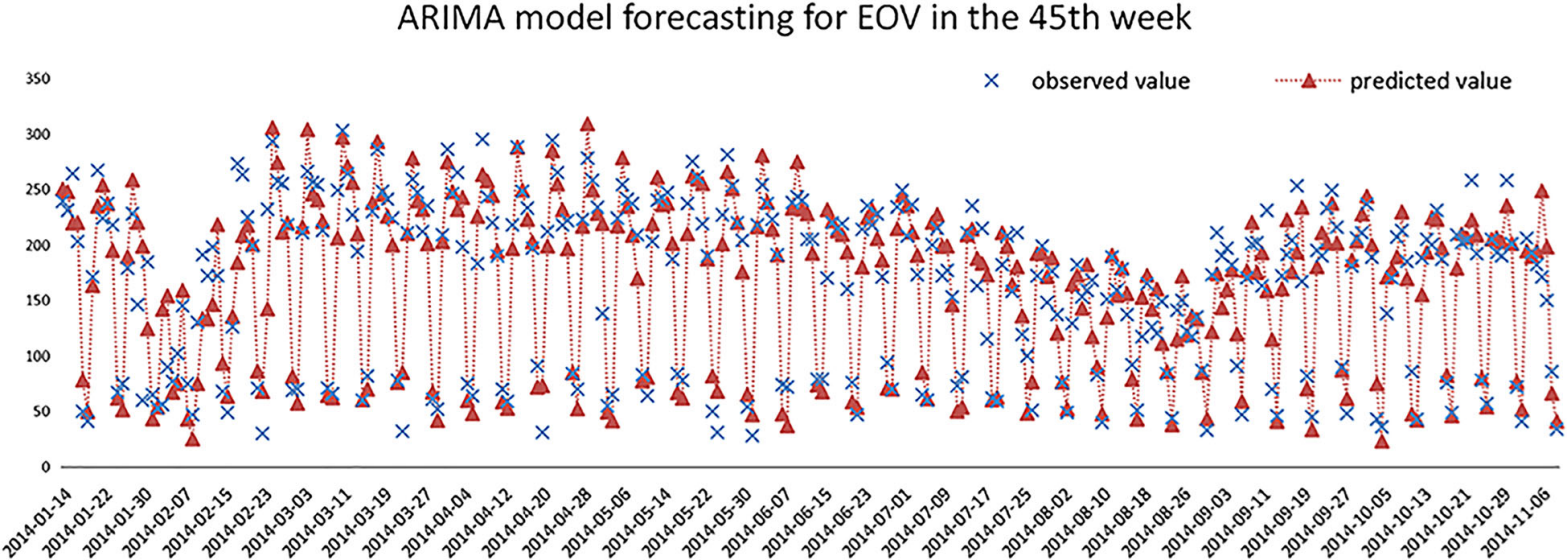
Fitted and predicted results using ARIMA model in endocrinology department

**Fig. 11:**
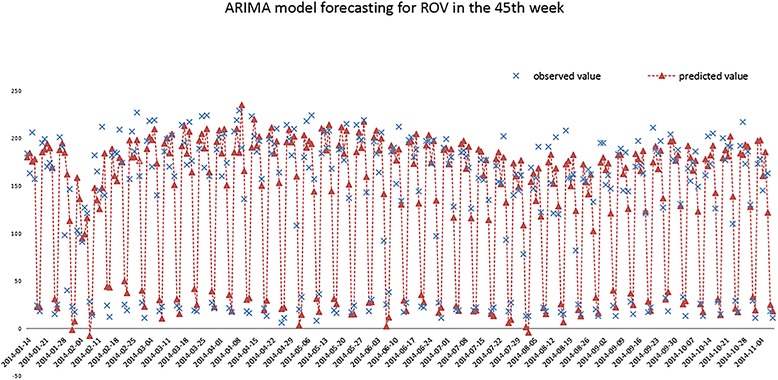
Fitted and predicted results using ARIMA model in respiratory department

The fitted models are used for short-term forecasting on the time series and the 7-day predicted values of the following week are obtained (Figs. 10, 11). The MAPE of the fitted and predicted values is 16.90 and 19.31% respectively in endocrinology department, while 25.32 and 25.44% in respiratory department.

### Building SES model

Aiming to capture the feature of the day of week effect, SES model is implemented for the time series of the day of the week (the same day in different weeks). The initial value for the smoothing $\widehat{{\mathrm{x}}_{1,\uptau}^2}$ and the smoothing constant α are determined using the method described in the above section. The predicted values are calculated using formula (3), and the MAPE of the fitted and the predicted values of EOV is 16.44 and 19.55% respectively, while the corresponding values of ROV is 16.56 and 21.90%.The predicted results from the two models in two departments are shown in Table 3.

**Table 3:** Forecasting performance comparison of two models during the 45th week

Time series data	Within a single week	Monday	Tuesday	Wednesday	Thursday	Friday	Saturday	Sunday	Forecasting performance MAPE
EOV	observed values	206	193	182	171	150	86	34	
	ARIMA model	194.40	189.47	194.27	248.46	197.90	66.11	41.00	19.31%
	SES model	196.76	195.68	190.00	256.56	193.76	76.33	46.28	19.55%
ROV	observed values	173	178	145	161	163	18	11	
	ARIMA model	197.32	197.88	160.23	174.97	121.83	24.85	18.03	25.44%
	SES model	186.12	196.28	177.78	185.36	126.76	27.32	13.61	21.90%

### Building the combinatorial model

The combinatorial model by combining SARIMA and SES model is established. Through the residual modification approach, the weighting coefficients for different days within a week are calculated with formula (4-6). The predicted and fitted values are presented in Tables 4 and 5, while residuals comparison between ARIMA, SES and combinatorial models are presented in Table 6. In turn, the prediction of the remaining 7 weeks can be computed using the same approach and process, through which the prediction results are finally acquired (Tables 7, 8, 9, 10).

**Table 4:** Fitting and forecasting performances of combinatorial model in endocrinology department during the 45th week

Within a single week	Monday	Tuesday	Wednesday	Thursday	Friday	Saturday	Sunday	Overall	Workdays	Weekends
Combinatorial model fitted	15.70%	10.39%	8.86%	17.55%	11.91%	16.21%	27.63%	15.46%	12.87%	21.92%
Combinatorial model predicted	5.09%	0.46%	5.75%	47.33%	30.45%	15.94%	29.04%	19.15%	17.82%	22.49%
Weighting coefficient l1	0.53	0.57	0.58	0.57	0.46	0.39	0.46			
Weighting coefficient l2	0.47	0.43	0.42	0.43	0.54	0.61	0.54

**Table 5:** Fitting and forecasting performances of combinatorial model in respiratory department during the 45th week

Within a single week	Monday	Tuesday	Wednesday	Thursday	Friday	Saturday	Sunday	Overall	Workdays	Weekends
Combinatorial model fitted	9.85%	8.86%	10.60%	7.73%	14.20%	28.69%	32.15%	17.14%	11.82%	30.37%
Combinatorial model predicted	10.96%	10.77%	16.37%	11.93%	23.69%	47.87%	38.05%	22.81%	14.74%	42.99%
Weighting coefficient l1	0.52	0.55	0.51	0.50	0.48	0.28	0.36			
Weighting coefficient l2	0.48	0.45	0.49	0.50	0.52	0.72	0.64

**Table 6:** Residuals comparison between ARIMA, SES and combinatorial model in the 45th week

Time series data	Model	ARIMA	SES	Combinatorial
EOV	Mean of residual	0.2358	−0.6678	−0.1994
	Standard deviation of residual	27.2980	30.9600	27.1130
ROV	Mean of residual	−1.8806	−0.7896	−1.2008
	Standard deviation of residual	20.4810	20.9640	19.4588

**Table 7:** Fitting and forecasting performances comparison of three models in two departments during 8 weeks (I)

Time series data	Fitted performance(MAPE)			Predicted performance(MAPE)		
	ARIMA	SES	Combinatorial	ARIMA	SES	Combinatorial
EOV	15.97%	16.72%	14.47%	11.77%	13.25%	10.61%
ROV	23.48%	16.55%	16.95%	15.26%	13.60%	13.49%

**Table 8:** Fitting and forecasting performances comparison of three models in two departments during 8 weeks (II)

Time series data	Prediction performance	Overall	Workdays	Weekends
EOV	ARIMA	11.77%	11.23%	13.13%
	SES	13.25%	12.57%	14.95%
	combinatorial	10.61%	10.19%	11.68%
ROV	ARIMA	15.26%	9.59%	28.49%
	SES	13.60%	9.78%	23.15%
	combinatorial	13.49%	9.51%	23.44%

**Table 9:** Residuals comparison between three models during 8 weeks

Time series data	Residuals comparison	ARIMA	SES	Combinatorial
EOV	Mean of residual	0.6625	0.4801	0.0504
	Standard deviation of residual	26.9557	30.1864	26.6696
ROV	Mean of residual	−1.5736	0.4905	−0.8760
	Standard deviation of residual	24.7444	24.5082	22.5943

**Table 10:** Weighting coefficient comparison between three models during 8 weeks

Time series data	weighting coefficient	Monday	Tuesday	Wednesday	Thursday	Friday	Saturday	Sunday	Overall	Workdays	Weekends
EOV	l1	0.52	0.57	0.57	0.57	0.47	0.38	0.45	0.51	0.54	0.42
	l2	0.48	0.43	0.43	0.43	0.53	0.62	0.55	0.49	0.46	0.58
ROV	l1	0.51	0.56	0.51	0.48	0.47	0.33	0.36	0.46	0.51	0.35
	l2	0.49	0.44	0.49	0.52	0.53	0.67	0.64	0.54	0.49	0.65

As seen above, in the two empirical studies, the result of analysis show the combinatorial model has superior prediction performance, with small mean of residual errors and residuals variance.

## Discussion

This paper examines the issue of forecasting the daily outpatient visits in China’s large hospital. Daily visits data of Chengdu outpatients in departments of endocrinology and respiratory in WCH over 2014 are used for forecasting. The proposed models, SARIMA model and SES model, can forecast daily outpatient visits in the short term well alone and there are considerable improvements in forecasting performance by combining the two complementary approaches together.

One important criterions of model selection is to select the appropriate method that has the best trade-off between the goodness-of-fit and the complexity of modeling (number of parameters). In this paper, we choose SARIMA and SES models to capture linear and nonlinear patterns, describe the features of autocorrelation, trend and cyclicity as well as the day of the week effect from different sub-time-series data respectively, and then establish a linear combinatorial model based on the above models through residual modification approach. All of the three models have good performance in forecasting accuracy. SARIMA model has predicted the daily number of EOV with a MAPE value of 0.1177 whereas it is 0.1325 for SES model, and 0.1061 for combinatorial model. And for the time series of ROV, the MAPE value of SARIMA model is 0.1526 whereas it is 0.1360 for SES model, and 0.1349 for combinatorial model. Moreover, the daily visits fluctuation of weekends series is relatively complex, which cannot be effectively extracted by single time series model. In our study, the combinatorial model is superior in modifying the extreme value of deviation in daily outpatient volume than SARIMA model,especially for the value of weekend’s data. For example, the results of weekend’s volume prediction show that MAPE reduces from 13.13 and 28.49% to 11.68 and 23.44% for EOV and ROV respectively. In general, the two single traditional models and the combinatorial model are simplicity of implementation and low computational intensiveness, whilst being appropriate for short-term forecast horizons over a large number of items.

As indicated both by the result of preliminary data analysis and the weighting coefficients, the fluctuations of daily outpatient visits within 1 week show different characteristics. Linear fluctuation is predominant from Monday to Thursday (l_1_ > 0.5) which can be better extracted by SARIAM model, while non-linear fluctuation is predominant from Friday to Sunday (l_2_ > 0.5) which can be better captured by SES model. That is to say, the fluctuation of daily outpatient visits from Monday to Thursday is more dominated by objective features of time series, while that from Friday to Sunday is dominated by subjective features (e.g., work shift schedule of doctors and healthcare seeking behaviors of patients). Moreover, the analysis result also show that most weighting coefficients of the combinatorial model range from 0.4 to 0.6, indicating that the linearity fluctuation (randomness and cyclicity) and non-linearity trends (the day of the week effect) captured by SARIMA model and SES model have a balanced impact on the final predictions of daily outpatient visits. In other words, for the forecasting of daily outpatient visits, both two features should be taken into account. Furthermore, different kinds of diseases have different volatility features. The results show the average value of weighting coefficient *l*_2_ of ROV (0.54) is bigger than that of EOV (0.49), which means that the non-linear fluctuation of ROV is more significant. In addition to the autocorrelation in daily time series data, the time series of ROV are more easily affected by uncertain factors. So, the prediction models of different diseases can be further optimized based on their own particular features and influence factors to increase forecasting accuracy.

The literatures show that there is no universal model applicable to any environment due to the inherent complexity of the time series data in the real world. The proposed model in this paper is designed to improve the forecasting accuracy from the data driven by incorporating the intrinsic characteristics of the historical time series data of hospital outpatient visits. It includes the randomness and cyclicity in the daily time series and the day of the week effect in weekly time series. According to this consideration, we adopt classical time series models and parametric estimation methods to ensure stability and convergence property. And based on this, we describe the combinatorial model to forecast the cross-sectional data for 7 consecutive days of daily outpatient visits over an 8-weeks period based on 43 weeks of observation data in two outpatient departments. As shown in the previous forecast results, the prediction performance of the combinatorial model is superior to the two single traditional models, as well as has the small mean of residual errors and better residuals variance. Thus it can be seen whether for the volume of ED visits or other time series data of healthcare system, the prediction model which is associated with effectively extracting meaningful features embedded implicitly in data is of great theoretical and practical significance. What’s more, by analyzing the research of outpatient visits forecasting, it shows that the combinatorial model can better observe and extract the general regularity of daily time series data in two different departments from a finite training sample size, while the ARIMA model has significant difference in feature identification among the two different diseases. And data collection and prediction of more kinds of disease should be discussed in the further research so as to validate the stable capability of the proposed model to discover the intrinsic laws of time series data.

Moreover, the forecasting horizon of 7 days for outpatient visits prediction in our paper is determined based on the current circumstances of healthcare system and hospital management requirement in China. It’s not hard to see China’s outpatient visit is more of a planned elective nature, and in light of the total outpatient amount controlling policy for large hospitals, the limited outpatient service resources and the weekly fixed team arrangement in most large hospitals, daily fluctuation within 1 week changes significantly compared to no evident difference on monthly or yearly fluctuation. Therefore, the prediction of daily outpatient visits about 1 week ahead has great realistic significance. Meanwhile, under such stable macro-environment (healthcare system) and micro-environment (hospital management requirement), the model can be applied to other large or medium scale hospitals where the daily time series of patient flow has the feature of randomness, cyclicity as well as the weekly pattern (the day of the week effect). Furthermore, medium and long term forecasting can be carried out based on the proposed combined model by adding enough new data sample until it can provide the relative complete information and features of the new time series.

The daily outpatient visits forecasting about 1 week ahead have practical implications in hospital operation management, not only contributing to guiding the long-term resource planning and scheduling in OD or other related sectors, but also helping to make tactical resolutions for short-term adjustment in special day. For an example, to deal with the problems of relative large prediction deviations for some certain day within a week, some operation measures such as appointment overbooking or adding capacities on the spot can be adopted in outpatient service management. The impact of non-linear variation of outpatient visits for Friday can be counteracted by reasonable management strategies, such as optimizing the work shift schedule of doctors who have strong effect of attracting patients and guiding the healthcare seeking behaviors of patients.

There are several limitations in this study. Firstly, the combinatorial model used in this paper is only for short-term forecasting from Monday to Sunday with 7 days in advance. More studies are required for moderate- and long-term forecasting of daily outpatient visits. Secondly, in addition to the inherent features of time series, other influence factors also come into play, such as the planned supply of outpatient resource and patients’ preference for certain doctors. The forecasting model can be optimized based on these features so as to achieve better prediction accuracy, especially for the daily visits prediction over the weekend. Thirdly, it is the lack of more discussions and analyses on alternative forecasting models, and reasons why they are not applicable. Fourthly, this paper only used outpatients’ visit data of two specific departments in one large hospital for forecasting, and data collection and prediction of more kinds of disease should be discussed in the further research, especially the chronic diseases.

## Conclusions

In conclusion, this paper contributes to exploration of prediction method to forecast outpatient visits in China’s large hospitals. We compared the forecast accuracy of a SARIMA model, a SES model, and that of a combinatorial model by combining SARIMA model with SES model for short-term daily outpatient visits forecasting. All of the selection models are relatively simple in terms of implementation and of low computational intensiveness, whilst being appropriate for short-term prediction. And compared simple models, the combinatorial model can more effectively extract depth information from a finite training sample size, and achieve better performance for predicting daily outpatient visits 1 week ahead, with lower residuals variance and relative small mean of residual errors which needs to be optimized deeply on the next research step. Also, the results can be used to support the decisions of outpatient resource planning and scheduling. It helps hospital managers to implement periodical scheduling of available resources on the basis of periodic features, as well as the proactive scheduling of additional resources based on the increasing regularity caused by the day of the week effect.

## Abbreviations

ACF: Autocorrelation function
ANN: Artificial neural network
BIC: Bayesian information criterion
ED: Emergency department
EOV: Endocrinology outpatient visits
ES: Exponential smoothing
MAPE: Mean absolute percentage error
MSARIMA: Multiplicative seasonal ARIMA
MSE: Mean square error
OD: Outpatient department
PACF: Partial autocorrelation function
ROV: Respiratory outpatient visits
SARIMA: Seasonal ARIMA
SES: Single exponential smoothing
SVM: Support vector machine
WCH: West China Hospital
